# Dominant Role of the Gut Microbiota in Chemotherapy Induced Neuropathic Pain

**DOI:** 10.1038/s41598-019-56832-x

**Published:** 2019-12-30

**Authors:** Chandran Ramakrishna, Jose Corleto, Paul M. Ruegger, Geoffrey D. Logan, Beth B. Peacock, Stacee Mendonca, Shanni Yamaki, Trinka Adamson, Richard Ermel, David McKemy, James Borneman, Edouard M. Cantin

**Affiliations:** 1 Department of Molecular Immunology, Beckman Research Institute of City of Hope, Duarte, CA 91010 USA; 2 Department of Biological Sciences and Section of Neurobiology, University of Southern California, Los Angeles, CA 90089 USA; 3 Department of Microbiology and Plant Pathology, University of California, Riverside, CA 92521 USA; 4 Center for Comparative Medicine, Beckman Research Institute of City of Hope, Duarte, CA 91010 USA

**Keywords:** Microbiology, Microbiology, Symbiosis, Symbiosis

## Abstract

Chemotherapy induced peripheral neuropathy (CIPN), a toxic side effect of some cancer treatments, negatively impacts patient outcomes and drastically reduces survivor’s quality of life (QOL). Uncovering the mechanisms driving chemotherapy-induced CIPN is urgently needed to facilitate the development of effective treatments, as currently there are none. Observing that C57BL/6 (B6) and 129SvEv (129) mice are respectively sensitive and resistant to Paclitaxel-induced pain, we investigated the involvement of the gut microbiota in this extreme phenotypic response. Reciprocal gut microbiota transfers between B6 and 129 mice as well as antibiotic depletion causally linked gut microbes to Paclitaxel-induced pain sensitivity and resistance. Microglia proliferated in the spinal cords of Paclitaxel treated mice harboring the pain-sensitive B6 microbiota but not the pain-resistant 129 microbiota, which exhibited a notable absence of infiltrating immune cells. Paclitaxel decreased the abundance of *Akkermansia muciniphila*, which could compromise barrier integrity resulting in systemic exposure to bacterial metabolites and products – that acting via the gut-immune-brain axis – could result in altered brain function. Other bacterial taxa that consistently associated with both bacteria and pain as well as microglia and pain were identified, lending support to our hypothesis that microglia are causally involved in CIPN, and that gut bacteria are drivers of this phenotype.

## Introduction

Chemotherapy has significantly increased the survival rate for multiple cancers due to improved detection and treatment. However, severe chemotherapy-induced side effects often limit the quality of life (QOL) of survivors and increased survivorship burdens the health care system. Chemotherapy-Induced Peripheral Neuropathy (CIPN) is the most severe and painful toxicity associated with the commonly used anti-cancer drugs, including taxanes, platinum compounds, and vinca alkaloids. CIPN is characterized by a broad range of symptoms, including twitching, pain, muscle weakness, numbness, burning and tingling^1^. Unfortunately, many patients respond poorly to conventional CIPN treatments, which can result in dose reduction that can reduce survival^2^. Furthermore, CIPN can persist long after treatment ceases and as it is often associated with cognitive impairment, anxiety, depression and fatigue this further degrades QOL^3,4^. Unfortunately, no agents have been identified to prevent CIPN, although many have been proposed and tested in clinical trials^2,5^.

Although the mechanisms of CIPN are unknown, the taxane, Paclitaxel and other common chemotherapy drugs are known to be neurotoxic, adversely enhancing the excitability of pain-sensing peripheral sensory neurons (nociceptors), producing reactive oxygen species that can activate pain receptors, disrupting mitochondrial electron transport and ATP production and disrupting microtubules resulting in peripheral sensory nerve fiber loss^6–9^. Evidence supporting a role for neuroinflammation in CIPN is accumulating, consistent with its role in models of neuropathic pain, diabetic neuropathy and traumatic brain injury^10–14^. Clarification of the mechanisms underlying CIPN is essential for development of new therapies that could increase survival across a wide range of cancers and improve QOL for cancer survivors.

Modern medicine is undergoing a renaissance, spurred by rediscovery of the importance of the gut microbiota for maintenance of human health and appreciation that disruption of the gut microbiota is associated with a plethora of human diseases^15^. The gastrointestinal tract is also a major target of chemotherapy toxicity, resulting in disruption of the gut mucosal barrier and development of mucositis^16,17^. Mounting evidence has linked the gut microbiota not only to chemotherapy efficacy, but also to nervous system toxicities, including development of peripheral neuropathy (PN), psychological and cognitive impairments^18–21^.

Inbred mouse strains are used extensively in pain research. However, they can exhibit significant differences in pain sensitivity with, for example, 129 strains being less sensitive than C57BL/6 (B6) strains^22,23^. Paclitaxel is a microtubule poison that is an essential chemotherapeutic regimen for breast, ovarian and other cancers, and it potently induces CIPN. We found that while Paclitaxel treated B6 mice developed pain, 129 mice did not, consistent with prior reports^24,25^. To determine involvement of the microbiota in this phenotypic difference in pain sensitivity, we compared Paclitaxel responses in B6 and 129 germ-free (GF) reciprocally transplanted mice. Remarkably, we found that the gut bacteria, rather than host genetics or physiology are the primary determinants of Paclitaxel induced pain. These important results provide a framework for future mechanistic studies to determine how the gut bacteria promote CIPN and that knowledge is expected to lead to novel, prebiotic, probiotic or synbiotic therapies for CIPN.

## Results

### A role for the microbiota in a mouse CIPN Model

**Figure 1:**
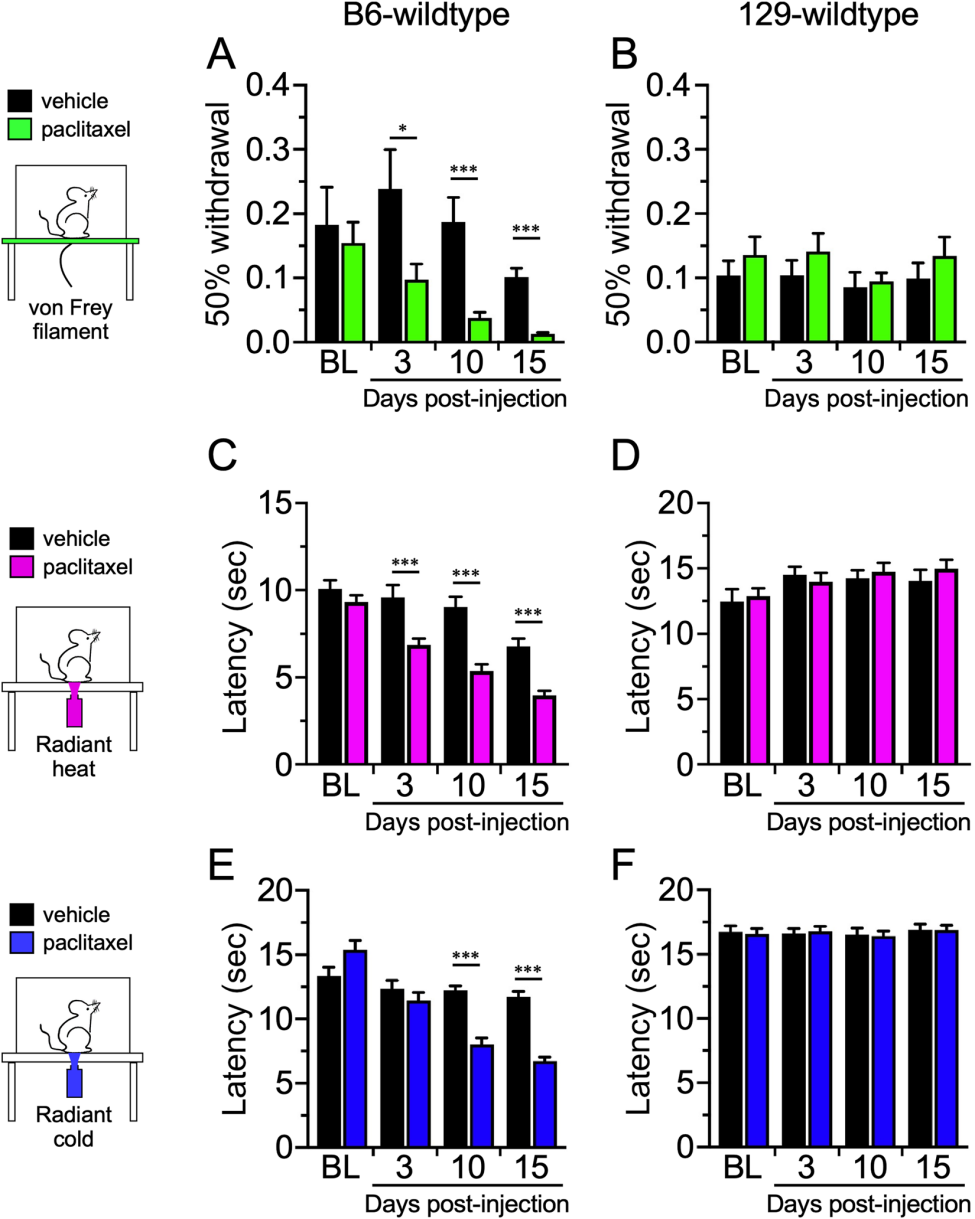
Paclitaxel-induced neuropathic pain is strain-dependent. Wildtype C57/Bl6 (B6) and Sv129 (129) mice injected with vehicle or Paclitaxel were tested for mechanical (**A,B**), heat (**C,D**), or cold (**E,F**) sensitivity on days 3, 10, and 15 days post injection. Paclitaxel-injected B6 mice show increased sensitivity by day 3 to heat and mechanical stimuli, and by day 10 for cold, compared to vehicle controls, whereas 129 mice were unaffected by Paclitaxel treatment at all stages. N = 16 for each (8 males and 8 females), *p < 0.05, ***p < 0.001.

To test the role of the gut bacteria in a mouse CIPN model, we first treated equal numbers of male and female wild type (WT) B6 and 129 mice with pharmaceutical-grade Paclitaxel given intra-peritoneally (ip) at 4 mg/kg on days 0, 2, 4, and 6, resulting in a final cumulative dose of 16 mg/kg. Control animals received an equivalent volume of the vehicle. To determine the induction of CIPN, we tested these animals sensitivity to thermal (heat and cold) and mechanical stimuli at 3, 10, and 15 days after the initial Paclitaxel injection^26,27^. B6 mice exhibited mechanical allodynia (Fig. 1A**)** and heat hyperalgesia (Fig. 1C) at day 3, 10, and 15 after Paclitaxel treatment, and cold allodynia by day 10 **(**Fig. 1E**)**. Additionally, the development of this form of CIPN in response to Paclitaxel treatment mirrors what we have previously reported for B6 mice treated with oxaliplatin^28^. Conversely,129 mice were refractory to Paclitaxel treatment in all three sensory modalities **(**Fig. 1B,D,F**)**. These data, encompassing multiple stimulus modalities, corroborate reports that 129 and B6 mice are respectively, relatively resistant and sensitive to neuropathic pain^22,23^.

**Figure 2:**
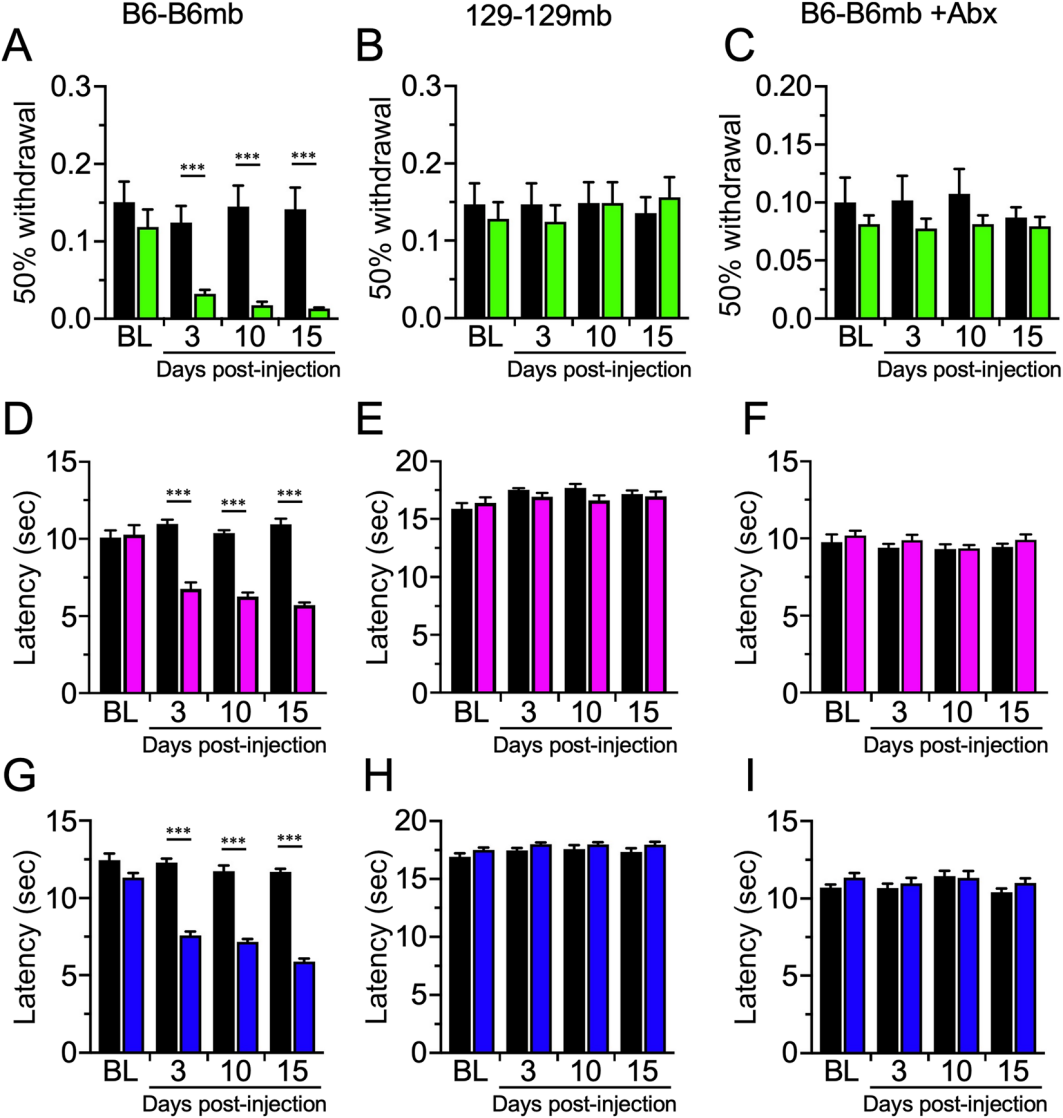
Paclitaxel sensitivity can be recapitulated in germ free mice by microbiota transfer. Germ-free B6 (**A,D,G**) and 129 mice (**B,E,H**) reconstituted with homologous fecal microbiota show similar sensitivity as wildtype mice post Paclitaxel injection. In germ-free B6 mice reconstituted with B6 microbiota, antibiotic treatment abolishes Paclitaxel-induced neuropathic pain (**C,F,I**). N = 16 for each (8 males and 8 females) except for 129-B6mb mice in which the n was 15 (8 males, 7 females), ***p < 0.001.

Next, we tested GF mice of both strains reconstituted with their own fecal microbiota. Consistent with the hypothesis that the microbiota determines development of CIPN, only reconstituted B6, but not 129 mice developed CIPN symptoms (Fig. 2), recapitulating phenotypic responses of the WT mice. Specifically, B6 mice reconstituted with the homologous microbiomes from B6 mice (B6-B6mb; Fig. 2A,D,G) and 129 mice reconstituted with 129 microbiomes (129–129 mb; Fig. 2B,E,H) mirrored, respectively, the B6 and 129 WT pain phenotypes in response to Paclitaxel treatment in all three stimulus modalities **(**Fig. 1**)**. Furthermore, treatment of B6-B6mb mice with an antibiotic cocktail to deplete the gut bacteria, precluded development of mechanical allodynia (Fig. 2C**)**, as well as increased heat (Fig. 2F) and cold sensitivity **(**Fig. 2I), consistent with prior reports that the gut bacteria are required for development of NP and CIPN symptoms^29,30^.

**Figure 3:**
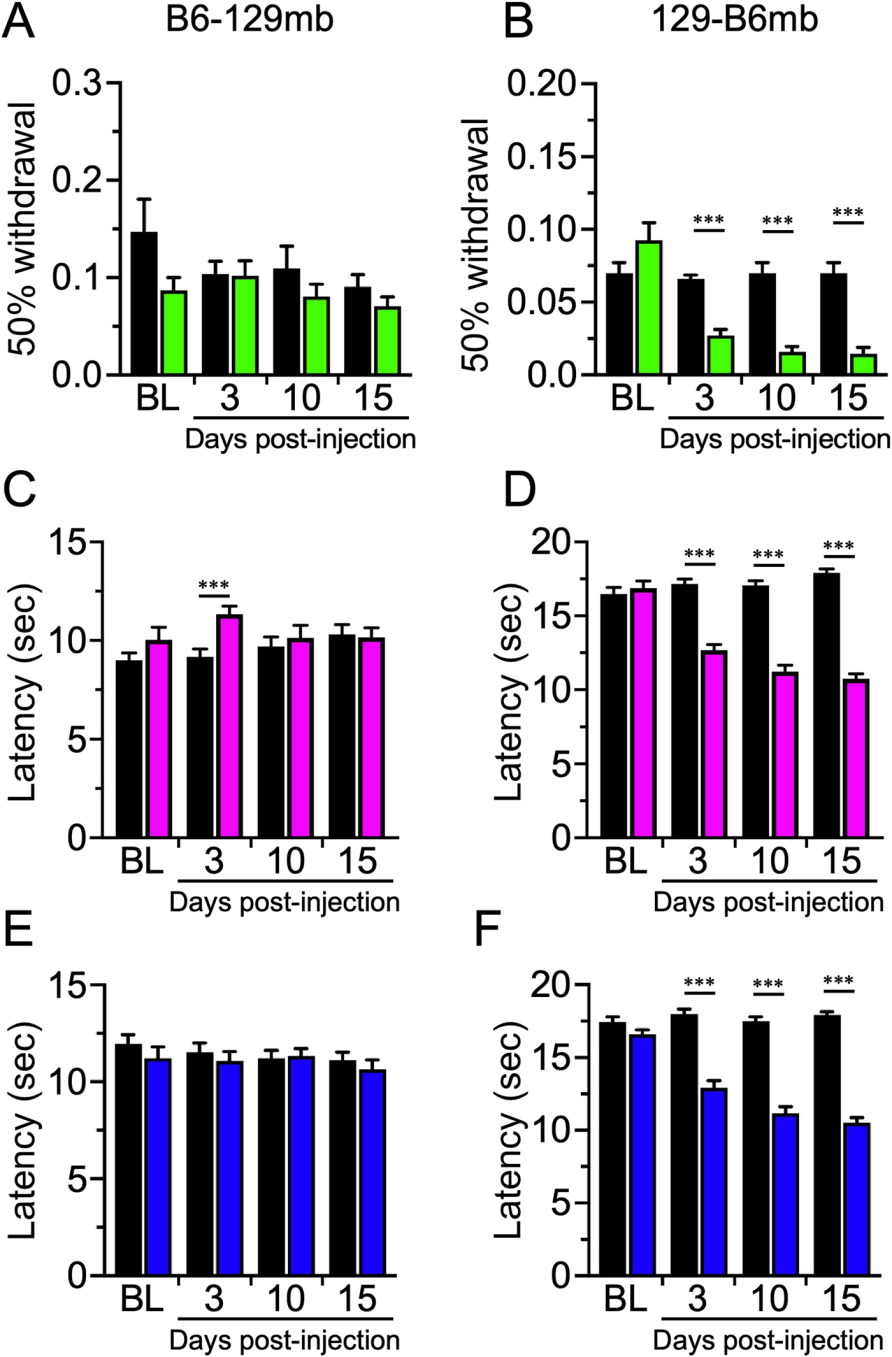
Paclitaxel-induced neuropathic pain is dependent on the microbiome. Germ free B6 and 129 mice reciprocally reconstituted with the opposite strain’s microbiota exhibit sensitivity that correlates with the microbiota of the donor strain and not the genetic background of the recipient mice. B6-129mb mice exhibited normal sensory acuity post-Paclitaxel (**A,C,E**) whereas 129-B6mb animals were neuropathic (**B,D,F**). N = 16 for each (8 males and 8 females), ***p < 0.001.

To gain insight into the relative importance of host genetics versus the microbiome in determining development of CIPN symptoms, we evaluated B6 and 129 mice reciprocally transferred with fecal microbiota from the other strain. Strikingly, 129 mice reciprocally transferred with microbiota from B6 mice (129-B6mb) mice displayed mechanical allodynia after Paclitaxel treatment (Fig. 3B), mirroring the phenotype of B6 wildtypes (Fig. 1A) and the fecal donor B6 mice **(**Fig. 2A**)**, while the B6-129mb Paclitaxel treated mice were resistant (Fig. 3A), phenocopying the WT 129 mice **(**Fig. 1B**)** and fecal donor 129 mice (Fig. 2B). Impressively, the pattern of phenotypic responses when these reciprocally transferred mice were tested for heat and cold sensitivity reflected that of the WT B6 and 129 mice, respectively (Fig. 3C–F). Of note, there was a slight yet significant decrease in heat sensitivity in B6-129mb mice at 3 days post Paclitaxel treatment, but this was not maintained after this initial time point (Fig. 3C) and not observed for either mechanical (Fig. 3A) or cold stimuli (Fig. 3E). Importantly, none of the CIPN symptoms were affected by sex (not shown), hence grouped data for 8 male and 8 female mice is presented unless otherwise stated. These data show that the gut bacteria are critical for induction of Paclitaxel neuropathy symptoms, validating our hypothesis that the gut bacteria are causally involved in CIPN.

### Inflammation in the spinal cord and brainstem of paclitaxel and vehicle treated mice

**Figure 4:**
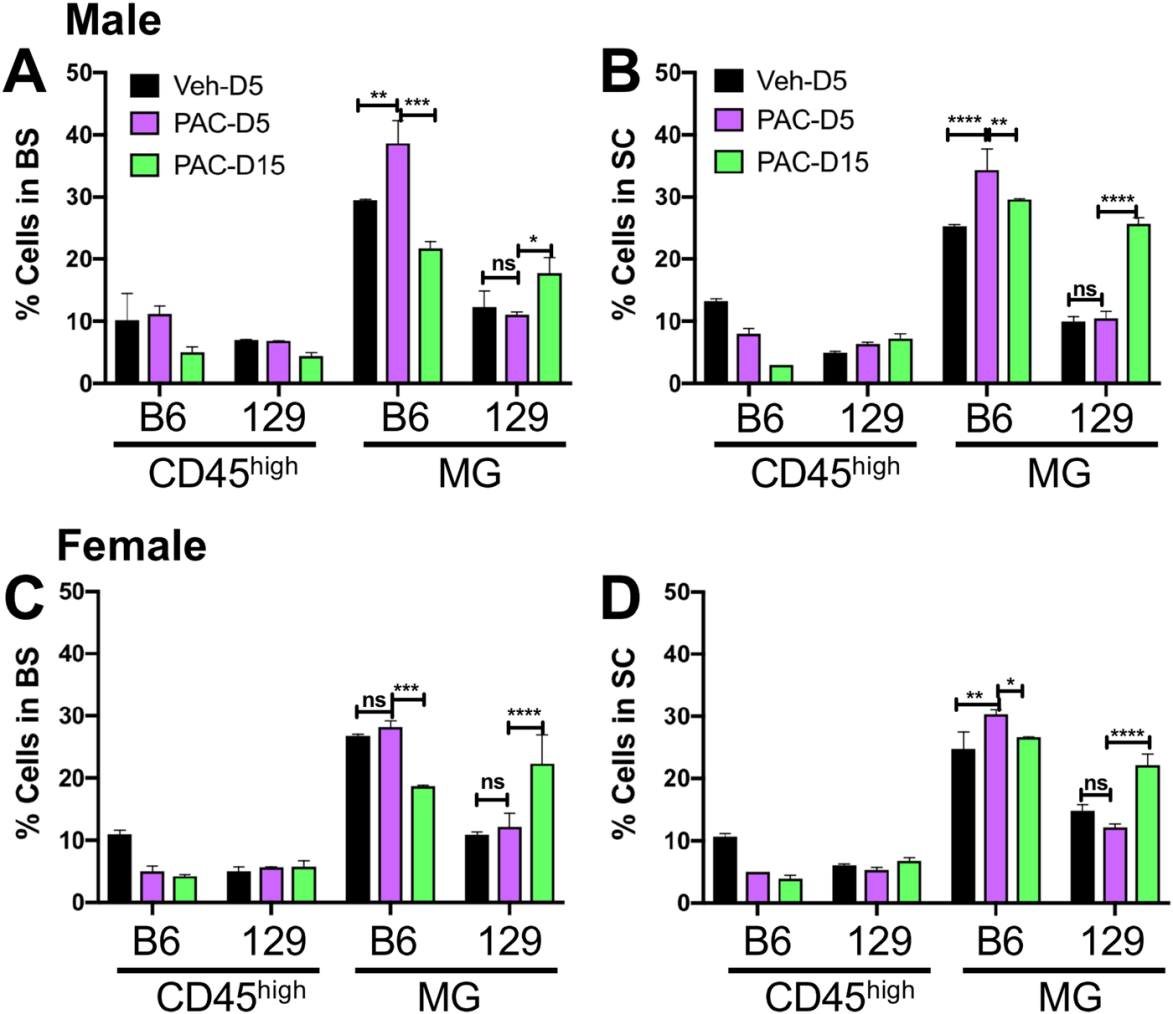
Microglial responses to Paclitaxel increased in B6 but not 129 WT mice. % CD45^high^ infiltrating leukocytes and CD45^low^ microglia (MG) in **(A,C)** brainstems (BS) and **(B,D)** spinal cords (SC) isolated on days 5 (D5) and 15 (D15) post treatment from **(A,B)** male and **(C,D)** female WT B6 and 129 mice treated with 4 doses of Paclitaxel (PAC: magenta: d5; green: d15) or vehicle (Veh: black). Data compiled from 2 experiments (n = 4 mice/group). *p < 0.05, **p < 0.01, ***p < 0.005, ****p < 0.0001, ns: not significant.

Evidence implicating inflammation in CIPN has increased and considering the critical role of the microbiota in development and regulation of the immune system, it was important to determine the effects of Paclitaxel on inflammation in the brainstem (BS) and spinal cord (SC). Flow cytometry analysis of mononuclear cells in the BS and SC of Paclitaxel- and vehicle-treated mice on day 5 and 15 post-treatment (pt) revealed only minimal and insignificant changes in CD45^high^ infiltrating leukocytes in Paclitaxel treated B6 and 129 mice. CD45^high^ cells in the BS and SC of B6 mice that developed pain in response to Paclitaxel were very low (<10%) and comparable to levels of infiltrating cells in 129 mice or vehicle treated B6 mice that did not develop pain (Fig. 4). However, in contrast to the paucity of infiltrating CD45^high^ cells, microglial (MG) accumulation (CD45^int^ CD11b^+^) was increased in the BS of male B6 mice and, particularly the SC of both male and female Paclitaxel treated B6 mice that developed pain, compared to vehicle treated mice that did not (Fig. 4). The gating strategy used to identify infiltrating cells and MG in the CNS is shown in S Fig. 1. Supplementary Fig. 1 also shows a lack of MHC II expression, which supports the lack of a role for T cell derived factors in pain, which requires IFNγ secretion by T cells. A tendency toward greater MG accumulation was seen at day 5 in BS and SCs of B6 male compared to female mice, but these sex differences were not significant. Interestingly, compared to B6 mice, MG accumulation was curtailed in the BS and SC of 129 mice that did not develop pain in response to Paclitaxel treatment and peak accumulation occurred on day 15 as opposed to day 5 for B6 mice (Fig. 4). By comparing MG accumulation in the SCs of Paclitaxel treated to that in vehicle treated mice at day 5, it was possible to discern a threshold of 1.30-fold that was exceeded in B6, but not 129 mice (Fig. 4B,D, Table 1). Microglial accumulation in the BS of Paclitaxel treated B6 mice did not meet this criterion consistently (Fig. 4A,C, Table 1), suggesting spinal MG are more important for induction of CIPN symptoms.

**Table 1:** The Microglial Index.

Figure 4*	129 WT	B6 WT	
Male BS	−1.12	1.31	
Male SC	1.05	**1.35**	
Female BS	1.11	1.05	
Female SC	−1.22	**1.32**	
Figure 5*	**129-129 mb**	**B6-B6mb**	**Abx B6mb**
Male BS	1.16	1.09	1.4
Male SC	1.16	**2.06**	1.29
Female BS	−1.11	−1.07	2.05
Female SC	1.05	**2.18**	−1.17
Figure 6*	**B6-129mb**	**129-B6mb**	
Male BS	1.19	1.58	
Male SC	1.23	**1.61**	
Female BS	1.28	1.33	
Female SC	1.07	**1.41**	

*Fold change in microglial populations within BS and SC of the different groups of mice treated with Paclitaxel compared to vehicle at day 5 after treatment – the Microglial Index, calculated using the means of data obtained from the figures indicated.

**Figure 5:**
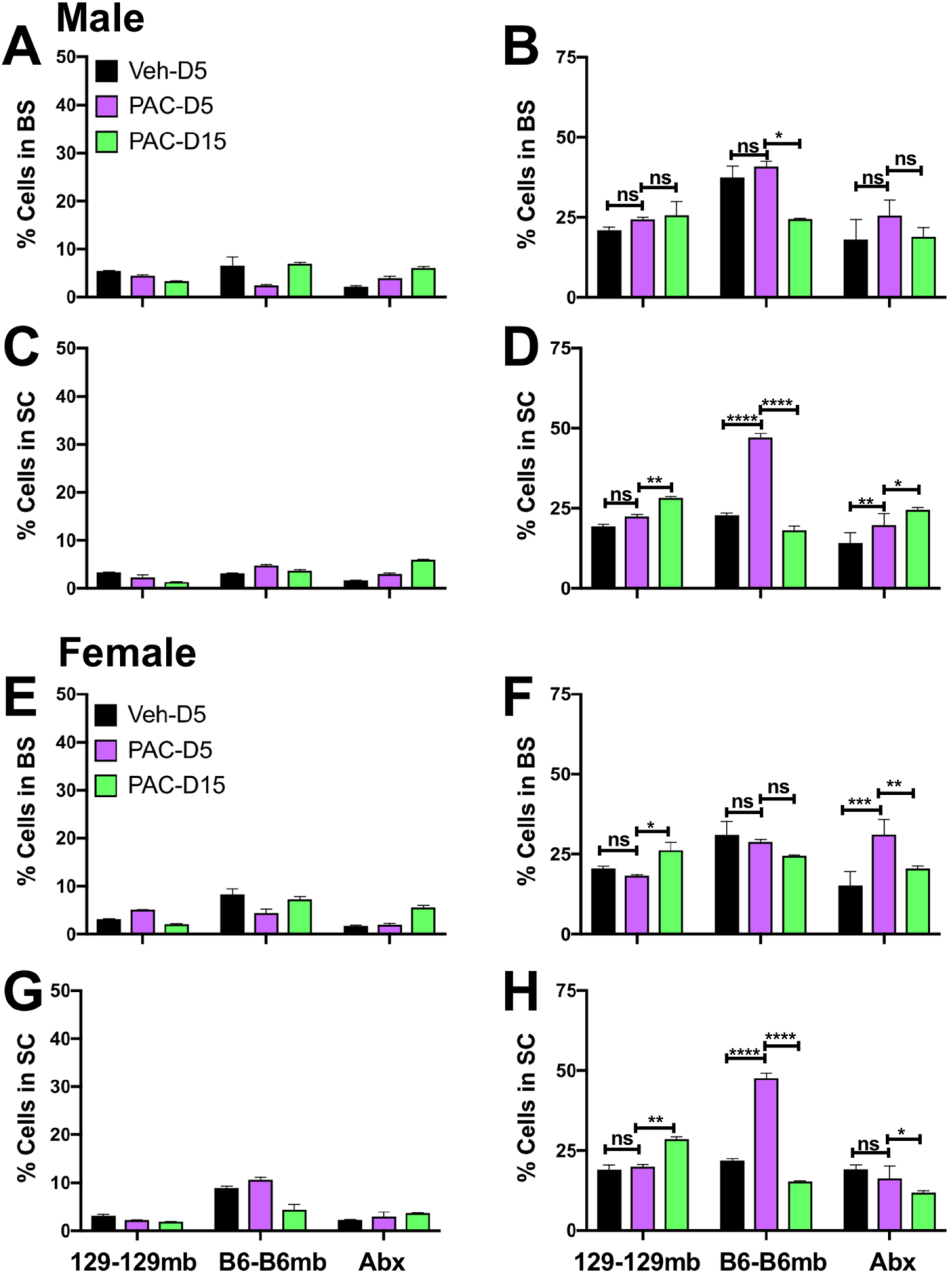
Microglial responses to Paclitaxel are induced by the B6 but not the 129 microbiota in homologously transplanted mice. (**A,C,E,G)** % CD45^high^ infiltrating leukocytes and **(B,D,F,H)** % CD45^low^ MG in **(A,B,E,F)** BS and **(C,D,G,H)** SC isolated on days 5 (D5) and 15 (D15) post treatment from **(A–D)** male and **(E–H)** female B6–B6 mb, 129–129 mb or antibiotic (Abx) treated B6–B6 mb mice treated with 4 doses of Paclitaxel (PAC: magenta: d5; green: d15) or vehicle (Veh: black). Data compiled from 2 experiments (n = 4 mice/group). *p < 0.05, **p < 0.01, ***p < 0.005, ns: not significant.

Analysis of inflammation in BS and SC of B6 and 129 GF mice harboring homologous or reciprocal microbiotas revealed that the MG accumulation induced by Paclitaxel treatment was profoundly influenced by the microbiome. As for CIPN symptoms, the pattern of CD45^high^ cell infiltration and MG proliferation in B6 and 129 GF mice reconstituted with their own microbiota phenocopied that of the corresponding WT mice (Fig. 5). Infiltration of CD45^high^ cells was minimal or absent in both groups of Paclitaxel and vehicle treated mice (Fig. 5A,C,E,G). Similar to WT mice, MG accumulation was increased in the SC of Paclitaxel treated compared to vehicle treated B6-B6mb, but not 129–129 mb mice (Fig. 5D,H). However, increases in MG accumulation were not observed in BS of B6-B6mb and 129–129 mb mice, emphasizing the importance of SC microglia (Fig. 5B,F). Importantly, antibiotic treatment to deplete gut bacteria markedly reduced MG proliferation in B6-B6mb mice to levels approximating that in 129–129 mb mice (Fig. 5B–H), concomitant with resistance to CIPN symptom development (Fig. 2C,F,I). It is noteworthy that the effects of gut bacteria depletion were more pronounced for MG in the SC than BS, for both male and female B6-B6mb mice (**compare** Figs. 5D and 5B**, and** 5H **and** 5F).

**Figure 6:**
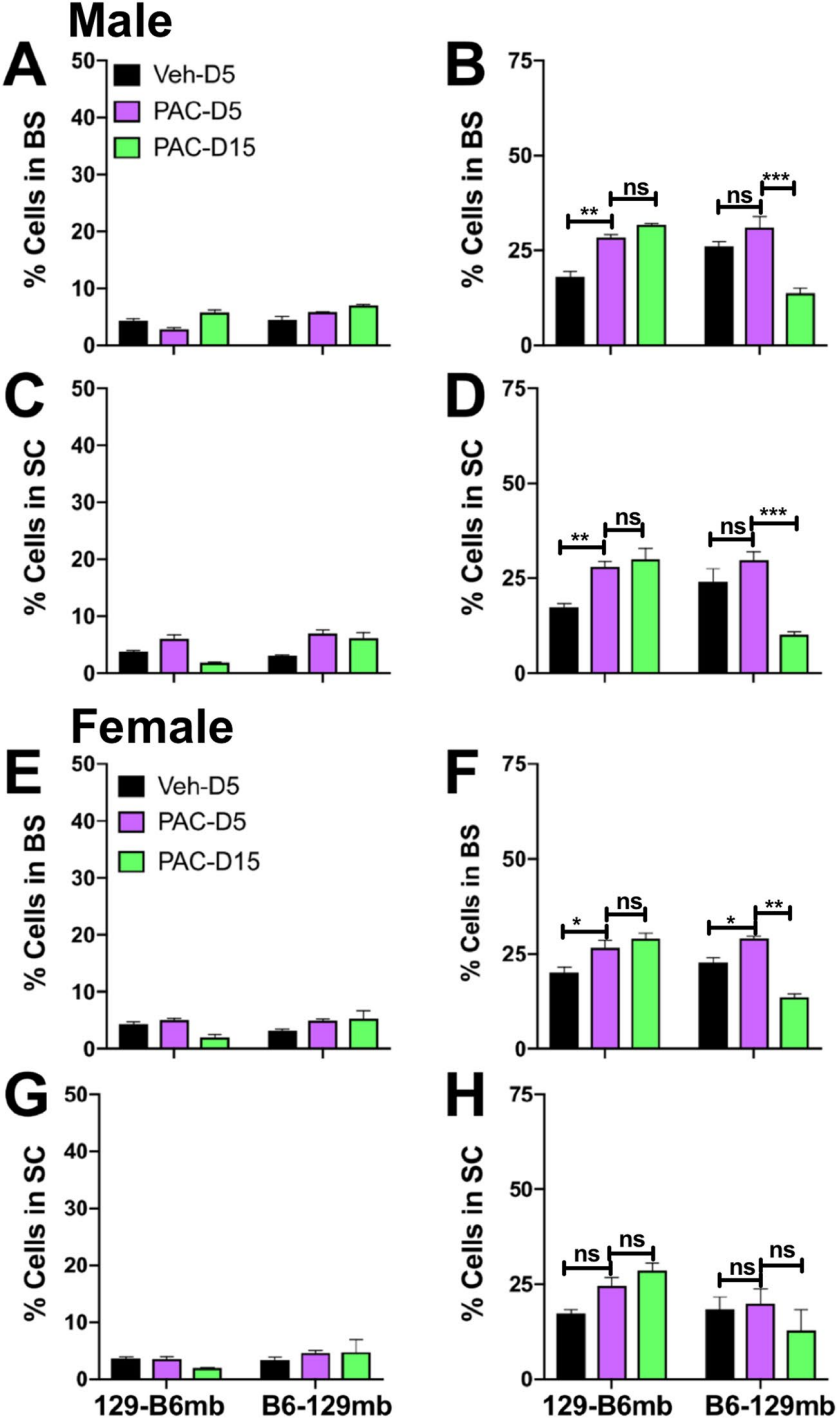
Microglial responses to Paclitaxel are induced by the B6 but not the 129 microbiota in mice heterologously transplanted mice. **(A,C,E,G)** % CD45^high^ infiltrating leukocytes and **(B,D,F,H)** %CD45^low^ MG in **(A,B,E,F)** BS and **(C,D,G,H**) SC isolated on days 5 (D5) and 15 (D15) post treatment from **(A–D)** male and **(E–H)** female B6–129 mb and 129-B6 mb mice treated with 4 doses of Paclitaxel (PAC: magenta: d5; green: d15) or vehicle (Veh: black). Data compiled from 2 experiments (n = 4 mice/group). *p < 0.05, **p < 0.01, ***p < 0.005, ns: not significant.

Consistent with results from WT B6 and 129 mice and mice derived by homologous FMT, infiltration of CD45^high^ cells into the SC and BS of mice derived by reciprocal FMT was minimal (Fig. 6A,C,E,G). However, the pattern of MG accumulation in SC and BS of Paclitaxel versus vehicle treated reciprocally transferred mice suggested that both the microbiome and host genetic background influenced this trait. Thus, Paclitaxel treated B6–129mb mice displayed reduced MG proliferation in SC and BS compared to 129-B6mb mice, relative to vehicle treated mice (Fig. 6B,D,F,H). Notably, CIPN symptom development (Fig. 3**)** occurred only in mice harboring B6mb that displayed an increase in MG accumulation in the spinal cord ≥ 1.3 fold at day 5 **(**Table 1). Whereas MG accumulation peaked at day 5 and then declined for WT and B6 homologous FMT mice, proliferation peaked at day 15 for 129-B6mb reciprocally transferred mice (compare Figs. 4–6). Similarly, MG proliferation peaked at day 5 before declining for B6-129mb reciprocal FMT mice, which is the pattern observed for 129 WT and homologous FMT mice. Thus, the timing of peak MG proliferation in responses to Paclitaxel treatment appears to be a genetically influenced trait. Though increased MG accumulation was noted in the BS of mice with B6mb compared 129 mb, a fold increase ≥ 1.3 was not a consistent finding as was the case for SC microglia (Table 1), which supports a role for spinal cord microglia being important for CIPN development, as has been reported for pain hypersensitivity after peripheral nerve injury and pain arising from stress^31,32^. Furthermore, increased MG proliferation ≥ 1.3 fold at day 5 appears to be an intrinsic property of the B6, but not the 129 microbiota (Table 1).

Interrogating chemokine and cytokine expression in the SC of B6-B6mb and 129–129 mb mice revealed that expression of most cytokine and chemokine genes was unchanged between the 2 groups, with only a modest upregulation of CCL2, IL-15 and Ppbp in B6mb mice compared to downregulation of CXCL13, CCL11, CCL17 and IL7 (S Fig. 2). It is conceivable that CCL2 induced the accumulation of CCR2 + microglia in B6-B6mb mice compared to 129–129 mb. However, although CCL2 is chemoattractant for monocytes, macrophages and lymphocytes these cells were not detected in the SC^33^. Since TLR4, and to lesser extent TLR2, have been implicated in inflammatory and neuropathic hypersensitivity^30,34,35^, we examined expression of TLR2 and TLR4 expression in the SC. Expression of TLR2 and TLR4 was detected in the SC at higher levels in B6-B6mb than 129–129 mb mice (S Fig. 3A). However, expression of TLR2 and TLR4 downstream signaling molecules was not detected using RT-PCR signaling arrays, discounting involvement of these TLRs in Paclitaxel induced pain (S Fig. 3B).

### Community analysis of bacteria from the different genotype-microbiota groups

**Figure 7:**
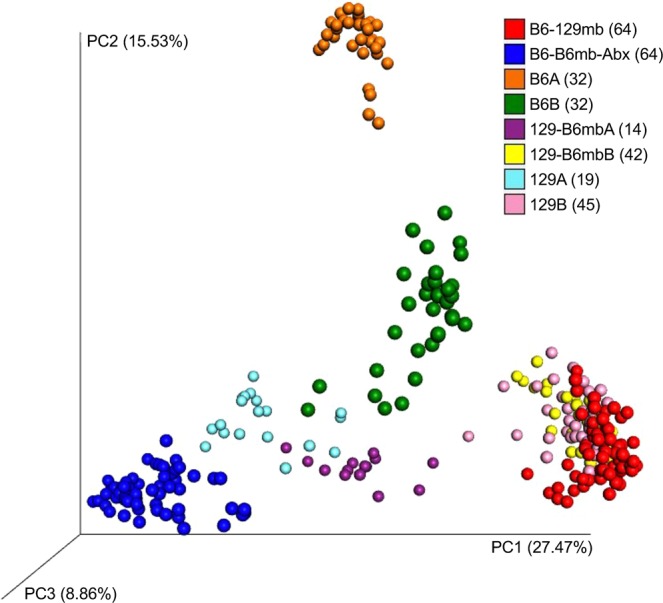
Community Analysis of the Fecal Bacteria from the Different Genotype-Microbiota Groups. This figure shows a principal-coordinates analysis (PCoA) of Hellinger beta diversity distance values generated from 16S rRNA gene sequences. The number of mice (n) in each genotype-microbiota group are shown in parentheses.

A beta diversity analysis of the fecal bacterial communities showed that three of the four main genotype-microbiota groups harbored two distinct communities, which led us to give them different names and to examine them separately (Fig. 7). For example, the B6 group was named B6A and B6B. See the Discussion section for possible reasons why this may have occurred. Adonis tests determined that all eight of the genotype-microbiota groups shown in the beta diversity figure (Fig. 7) were different from each other (P < 0.001). We also note that the pain phenotypes from each of these split groups were not statistically different (results not shown). Given the relatively low number of mice in 129 A and 129-B6mbA, some of the treatment types (Day 0, Day 10, Pac, Veh) had less than 2 mice. Accordingly, 129 A and 129-B6mbA were not included in the subsequent microbiome analyses.

### Heat maps of the abundant bacterial Operational Taxonomic Units (OTUs)

We examined the most abundant fecal bacterial OTUs in two ways to address two different hypotheses. By examining the samples from the mice before Paclitaxel administration, we addressed the hypothesis that it is the initial microbiota (before Paclitaxel was administered) that drives the pain phenotypes. By examining the Paclitaxel-induced changes in the microbiota, we addressed the hypothesis that it is the chemotherapy-induced changes in the microbiota that drives the pain phenotypes.

**Figure 8:**
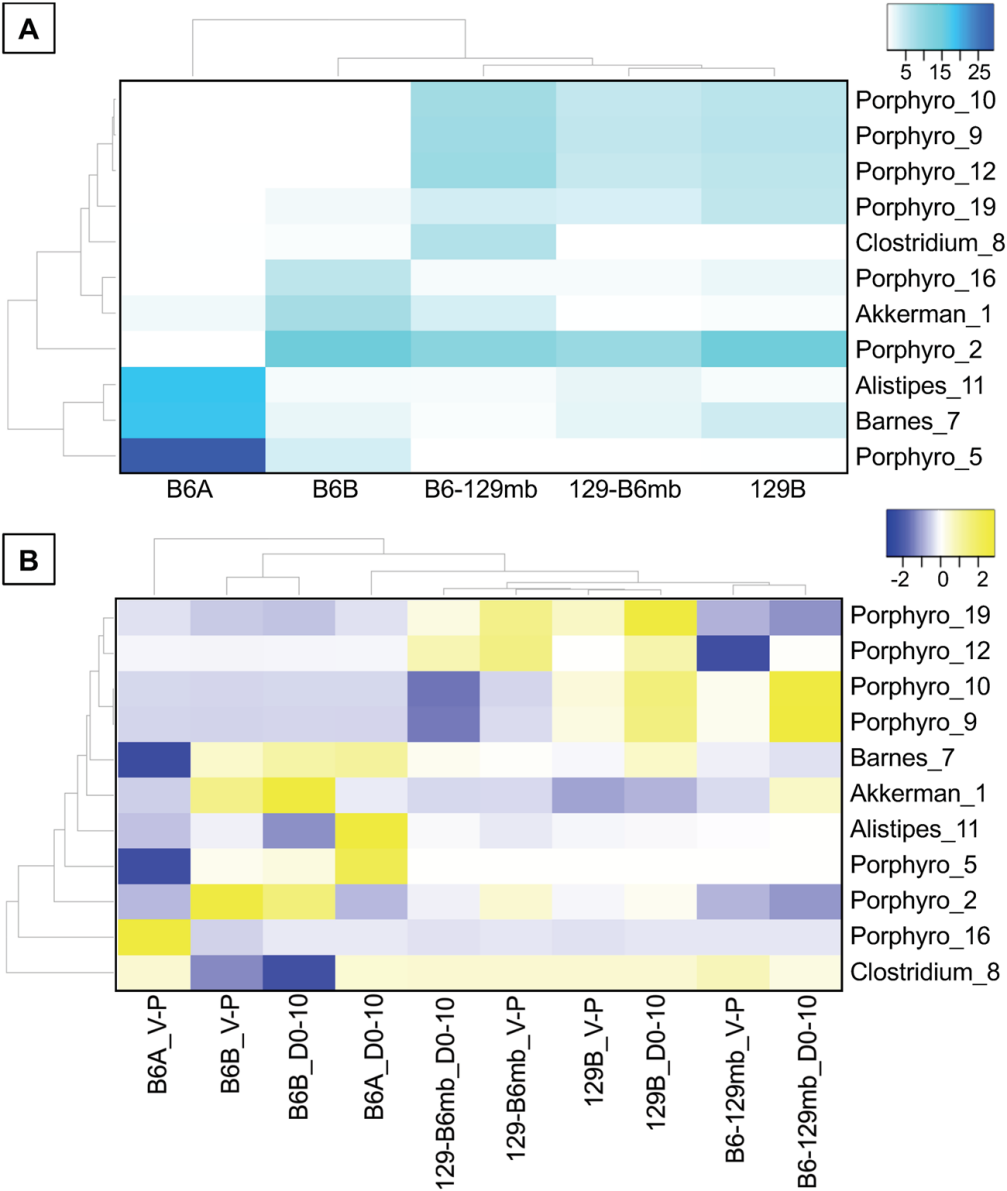
Heat Maps of the Abundant Bacterial OTUs. Heat Maps of the Abundant Bacterial OTUs. (**A**). Heat map of the abundant fecal bacterial OTUs from the genotype-microbiota mouse groups at Day 0 (before Pac was administered). Values in the cells are relative abundances of the bacteria, and only those OTUs with greater than 2% average relative abundance are shown. (**B**) Heat map of the Pac-induced differences in the abundant fecal bacterial OTUs from the genotype-microbiota mouse groups. Values in the cells are the differences in mean relative abundances of the bacteria where: V-P = Veh minus Pac and D0-10 = Pac at D0 minus Pac at D10. Heat maps were clustered on both axes. Number of replicates for each genotype-microbiota group are shown in Fig. 7. Bacterial taxa abbreviations are: Porphyro, *Porphyromonadaceae*; Clostridium, *Clostridium aerotolerans*; Akkerman, *Akkermansia muciniphila*; Alistipes, *Alistipes onderdonkii*; Barnes, *Barnesiella*; and the numbers following the underscores indicate the OTU numbers.

A heat map of the most abundant fecal bacteria in the genotype-microbiota groups at Day 0 shows several distinct patterns (Fig. 8A). Although B6A and B6B mice exhibited similar pain phenotypes (results not shown), they harbored different relative abundances of many of the most abundant fecal bacterial OTUs. While 129-B6mb mice exhibited a different pain phenotype than the two mouse groups harboring the 129-microbiota (129B and B6-129mb) (Figs. 2 and 3), these three groups clustered together suggesting that the 129 genotype has a stronger influence on fecal bacteria than the B6 genotype.

A heat map of the Paclitaxel-induced differences in the most abundant fecal bacteria showed some patterns among the genotype-microbiota groups (Fig. 8B), although they appeared to be less distinct than those observed in the Day 0 heat map (Fig. 8A). Two types of Paclitaxel-induced differences in the bacteria occurred, the first between the Veh and Paclitaxel treatment groups 10 days after Paclitaxel administration (V-P). The second was between the two Paclitaxel treatment groups – before administration and 10 days after Paclitaxel administration (D0-D10). These results showed that the two different ways to examine Paclitaxel-induced differences did not produce consistent results for all bacterial OTUs.

### Bacterial OTUs whose abundances were different among the genotype-microbiota and treatment groups

To provide a statistical and more detailed depiction of the heat map results, we examined the abundant fecal bacterial OTUs that were different among the genotype-microbiota and treatment (Day 0, Day 10, Paclitaxel, Veh) groups. For the Day 0 only analyses, out of the 110 possible pairwise comparisons of the eleven OTUs, 91 were statistically different (FDR-adjusted P value < 0.05, Supplemental Table 2). For the Paclitaxel-induced differences, out of the 110 possible pairwise comparisons of the eleven OTUs, 10 were statistically different (Supplemental Table 3). These results corroborate our visual assessment of the heat maps that suggested there were more coherent patterns in the Day 0 only analysis than the Paclitaxel-induced differences analysis.

**Figure 9:**
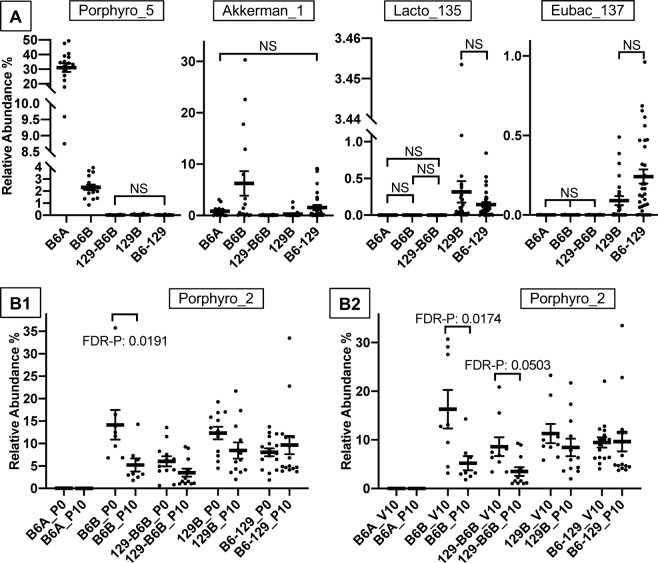
OTUs Whose Abundances Were Different Among the Genotype-Microbiota and Treatment Groups. (**A**) Example OTUs that showed some of the common patterns observed in the Day 0 heat map (FIGURE A). (**B**) Example OTU that showed patterns observed in the Pac-induceddifferences heat map (FIGURE B). In each of the A Figures, all possible pairwise comparisons of the genotype-microbiota groups are different (FDR-adjusted P value < 0.05) unless otherwise indicated by NS (not significant) or by a P value (non-FDR-adjusted). In each of the B Figures, only the statistically significant comparisons are shown. For both Figures A and B, these rules only apply to those pairs indicated by the vertical lines of the brackets; for example, for Porphyro_5, the only pair that is not statistically significant is 129-B6B and B6-129. Bacterial taxa abbreviations are:Akkerman, Akkermansia muciniphila; Eubac, Eubacterium siraeum; Lacto, Lactobacillus intestinalis; Porphyro, Porphyromonadaceae; and the numbers following the underscores indicate the OTU numbers. Genotype-microbiota and treatments abbreviations are: B6-129, B6-129mb; 129-B6, 129-B6mbB; P0, Pac at Day 0; P10, Pac at Day 10; V0, Veh at Day 0; V10, Veh at Day 10. Means are the thick horizontal lines, error bars are standard error, and the replicate samples are the dots.

Next, we made figures of several OTUs that showed some of the common patterns observed in the Day 0 only heat map, which are the fecal bacteria before Paclitaxel was administered (Fig. 9A). In each of these figures, all possible pairs of the genotype-microbiota groups are different (FDR-adjusted P value < 0.05) unless indicated by NS (not significant). In addition, these rules only apply to those pairs indicated by the vertical lines of the brackets; for example, for Porphyro_5, the only pair that is not statistically significant is 129-B6B and B6-129.

Example OTUs that were different in their relative abundances in the mouse groups harboring the B6 microbiota compared to those harboring the 129 microbiota before Paclitaxel was administered (Day 0) were Lacto_135 (*Lactobacillus intestinalis*) and Eubac_137 (*Eubacterium siraeum*) (Fig. 9A). Example OTUs that support the heat map observation that B6A and B6B harbor different microbiota were Porphyro_5 (*Porphyromonadaceae*) and Akkerman_1 (*Akkermansia muciniphila*). Additional examples are shown in S Fig. 4.

Next, we made figures of OTUs that showed patterns observed in the Paclitaxel-induced-differences heat map (Fig. 9B). In each of these figures, only the statistically significant comparisons of the abundant bacteria are shown. Two types of Paclitaxel-induced differences (or comparisons) in the fecal bacteria are shown in these figures. The first comparison was between before Paclitaxel was administered and 10 days after Paclitaxel was administered (D0-D10; Fig. 9B1). The second comparison was between the Veh and Paclitaxel treatment groups 10 days after Paclitaxel administration (Fig. 9B2). OTU Porphyro_2 (*Porphyromonadaceae*) is an example where Paclitaxel decreased its relative abundances in two B6 microbiota groups – B6B and 129-B6B (Fig. 9B1,2). Conversely, Paclitaxel had relatively little impact on the populations of OTU Porphyro_2 in the two 129 microbiota groups. Similarly, OTU Porphyro_16 (*Porphyromonadaceae*) exhibited a Paclitaxel-induced decrease in its relative abundance in the B6A group but not in the 129 microbiota groups (S Fig. 5). Given these results, OTUs Porphyro_2 and Porphyro_16 exhibit trends consistent with pain-inhibiting bacteria. Additional examples are shown in S Fig. 5.

### Bacterial OTUs whose abundances correlated with microglia levels

To examine the relationships between the abundant fecal bacterial OTUs and microglia, we performed correlation analyses. These analyses identified several correlations that were consistent with the association results between both bacteria and pain as well as between microglia and pain (described above). For example, two of the putatively pain-inhibiting OTUs (Porphyro_2 and Porphyro_16) – shown in Fig. 9B1,2 and S Fig. 5 – exhibited negative correlations with microglia in the B6 microbiota groups but not in the 129 microbiota groups (S Fig. 6). These results are consistent with the hypothesis that microglia are causally involved in Paclitaxel-induced pain, and that gut bacteria are drivers of this phenotype.

## Discussion

There are several reports that 129 mice present significant, and in some cases, extreme phenotypic differences in several measures of analgesia, nociception and hypersensitivity induced by different stimuli, compared to the C57BL/6 mice^24,36,37^. We therefore determined the response of these strains to Paclitaxel, a frontline chemotherapeutic drug that is a major cause of CIPN. We observed extreme phenotypes for mechanical and cold allodynia as well as heat hyperalgesia, with B6 mice being sensitive, while 129 mice were resistant to Paclitaxel induced pain. Antibiotic depletion of the gut bacteria impeded development of all three sensory modalities in B6 mice, revealing an essential role of the microbiota for Paclitaxel induced PN symptoms. Gut bacteria have also been reported to be essential for oxaliplatinin induced mechanical alloydynia^30^ and for development of inflammatory pain^29^, which suggests gut bacteria may have an important role enabling diverse pain conditions. Analysis of B6 and 129 mice derived by reciprocal FMT revealed that Paclitaxel-induced pain developed only in mice harboring B6mb but not 129 mb. This remarkable result shows that Paclitaxel induced pain is strictly determined by the gut microbiota rather than host genetics or physiology.

Analysis of spinal cord and brainstem in Paclitaxel and vehicle treated mice showed that inflammatory infiltrates were largely absent, contrary to expectations based on prior reports of Paclitaxel-induced neuroinflammation in the DRG and spinal cord involving macrophages/monocytes and/or microglia^38–40^. However, these studies used immunostaining that could not rigorously discriminate macrophages and microglia, as they employed markers expressed by both cell types. However, more rigorous flow cytometery analysis of single cell suspensions showed that CD45^hi^ infiltrating cells were largely absent from BS and SC of vehicle and Paclitaxel treated mice. In contrast, increased microglial accumulation was observed in the SC, and to a lesser extent BS, in response to Paclitaxel treatment of B6mb but not 129 mb mice that developed or resisted Paclitaxel induced pain, respectively. Microglial expansion, which was more evident in male than female mice, was reduced in mice with 129 mb and abrogated by Abx treatment of B6mb mice, implicating microglia in Paclitaxel-induced pain, as both of these groups of mice were pain free.

The microbiota are critical for controlling microglial maturation and function as shown by antibiotic treatment of adult mice resulting in impaired maturation and innate immune functionality of these brain resident immune cells^41,42^. Recent data supports microglia-microbiome connectivity having a pivotal role in orchestrating the gut-brain axis that is causally implicated in various neurodegenerative and neurobehavioral conditions^43,44^. Our data provide compelling evidence implicating spinal microgliosis as being causally involved in Paclitaxel-induced pain, adding to reports supporting causal involvement of the microglia in peripheral nerve injury and stress induced pain, as well as neuroinflammatory pain^13,31,32,45^. Although microgliosis was more prominent in males, we did not observe sex differences in the three Paclitaxel induced sensory modalities, possibly because the mouse group numbers were too low.

The beta diversity results from community analysis of fecal bacteria from the different genotype-microbiota groups showed that there were two distinct subgroups for three of the four main genotype-microbiota groups. The differences in the B6 communities likely resulted from The Jackson Laboratories sending us two batches of mice, each of which came from a different vivarium. However, we could not identify variables that could explain the splitting of the 129 and 129-B6mb groups. Since the groups with common microbiota – for example, B6A, B6B, and 129-B6mb – had similar pain phenotypes, we posit that there are different microbes or microbial consortia in these subgroups driving the pain resistant or pain sensitive phenotypes.

To address our hypothesis that gut bacteria are causally invovled in the pain phenotypes, we examined the abundant fecal bacterial OTUs that were different among the genotype-microbiota and treatment groups in two ways. Example OTUs supporting the hypothesis that the initial microbiota (Day 0) are the drivers of the pain phenotypes are the *Lactobacillus intestinalis* OTU (#135) and the *Eubacterium siraeum* OTU (#137). Given their population trends, we posit that they are inhibitors of the pain phenotype. An example supporting the hypothesis that the Paclitaxel-induced changes in the gut microbiota are the drivers of the pain phenotypes is the *Porphyromonadaceae* OTU (#2). The population trends of this bacterium suggest that it also may be driving the pain inhibiting phenotype.

A search of the literature determined that only two of our abundant gut bacteria have been previously associated with pain. In our study, the only statistical difference in the Paclitaxel-induced analyses of the *Alistipes* OTU was an increase from Paclitaxel Day 0 to Pac Day 10 in the B6B group. This trend suggests that this bacterium might cause pain, which is consistent with two prior studies^46,47^. In one of these studies, chronic fatigue syndrome (CFS) was examined in humans. Here, the relative abundance of *Alistipes* was positively associated with CFS, and the criteria for a CFS diagnosis included four different types of pain: lymph node pain, muscle pain, joint pain and headaches of a new or different type^47^.

In the Paclitaxel-induced analyses of our study, there were decreases in the relative abundance of the the *A. muciniphila* OTU in the B6B microbiota from Paclitaxel Day 0 to Paclitaxel Day 10 and from Veh Day 10 to Paclitaxel Day 10, which suggests that this bacterium might inhibit pain. This potential phenotype is consistent with three prior studies^48–50^. In one of these studies, IBS was studied in humans^49^. Here, fecal microbiota transfers ameliorated abdominal pain and were associated with an increase in *A. muciniphila*^49^. In our study, for the Paclitaxel-induced analyses of the 129-microbiota group, there were two contradictory changes – one suggesting a potential pain-inhibiting role for the *Akkermansia* OTU and the other suggesting a potential pain-causing role of this bacterium. This result suggests that for the 129-microbiota group, this bacterium may not play a role, or at least not a consistent role, in pain perception.

Taken together, these results support our hypothesis that chemotherapeutic agents such as Paclitaxel decrease the amounts of beneficial bacteria such as the *A. muciniphila* OTU, which has been shown to promote barrier functioning^51^. This in turn supports our global hypothesis where chemotherapy causes barrier dysfunction resulting in increased systemic exposure to bacterial products and metabolites, which promotes systemic inflammation that drives pain sensitivity. Linking these events together are OTUs such as *Porphyromonadaceae* 2 and 16, which exhibited consistent associations between both bacteria and pain as well as between microglia and pain, supporting our hypothesis that microglia are causally involved in Paclitaxel-induced pain, and that gut bacteria are drivers of this phenotype.

## Methods

### Mice

Animal care and use was approved by the City of Hope Institutional Animal Care and Use Committee and conformed to recommendations of the NIH Guide for the Care and Use of Laboratory Animals. Wildtype (WT) B6 (C57BL6/J) and 129 WT (129S6/SvEvTac) obtained from Jackson Laboratories (Bar harbor, Maine) and Taconic (Hudson, NY) respectively, were bred in the Animal Resources Center at City of Hope. B6 germ free (GF) mice were obtained from Dr. Sarkis Mazmanian’s gnotobiotic colony at Caltech (Pasadena, CA), while the 129 GF mice were obtained from The National Gnotobiotic Rodent Resource Center (NGRRC) at UNC-Chapel Hill and North Carolina State University. Immediately upon arrival male (n = 6) and female (n = 2) GF WT B6 and 129 mice were gavaged with fecal slurries prepared from WT B6 (B6mb) and 129 (129 mb) SPF mice to generate B6 and 129 mice colonized with homologous or heterologous microbiota resulting in the following mouse lines: B6-B6mb, B6-129mb, 129–129 mb and 129-B6mb. The GF mice were gavaged with 100 μl of fecal slurry (50 mg/ml) prepared by dispersing fecal pellets in PBS + 1.5% Sodium bicarbonate buffer by passage through a 25-guage syringe. Successful colonization was confirmed by plating fecal slurries on appropriate agar plates. Fecal pellets were vortexed thoroughly and then plated on Blood Agar Plates (BAP), MacConkey Agar (MAC), pre-reduced CDC Anaerobic Agar, and Laked Blood Agar with Kanamycin and Vancomycin (LKV) in duplicates with 100-µl of each fecal slurry placed in aerobic and anaerobic incubators. The aerobic plates were read after 24 hours of incubation and anaerobic plates after 48 hours of incubation. Breeding colonies for each of the FMT lines were established and maintained by the Center for Comparative Medicine Colony Management Program. Mouse lines were housed in individually ventilated autoclaved cages supplied with autoclaved bedding, irradiated food, and reversed-osmosis water and maintained within a Specific Pathogen Free (SPF) breeding barrier. All animal transfers and manipulations were performed within animal transfer hoods using aseptic techniques and SPF barrier standard operating procedures. Offspring from these lines were used in all subsequent experiments.

### Antibiotic depletion of gut bacteria

B6-B6mb mice were treated with an antibiotic cocktail (Abx) containing neomycin (1 g/L), ampicillin (1 g/L), and vancomycin (0.5 g/L) and metronidazole (1 mg/ml) to deplete gut bacteria^52–54^. The antibiotics were thoroughly mixed into Medigel Sucralose Cups (Clear H_2_O), a non-wetting sucralose flavored, low calorie water gel designed to mask the medication taste so as to achieve efficient consumption by research animals. One Medigel Cup was used per cage of 4 mice, with new cups delivered on Mondays, Wednesdays and Fridays. The mice were treated with Abx for 14 days, which is sufficient to reduce gut bacteria levels to those in GF mice^52^.

### Behavioral analyses

All procedures and tests were approved by the University of Southern California Institutional Animal Care and Use Committee and conducted in accordance with the recommendations of the International Association for the Study of Pain and the NIH Guide for the Care and Use of Laboratory Animals. Vehicle used throughout this study was Cremophor EL: ethanol diluted with saline. The experimenter was blinded to if the animals were treated with Paclitaxel or vehicle. All results from behavioral assays were expressed as mean +/− SEMs for each group. The sample size in each experiment was based on our previous studies on similar experiments^26^. Behavioral data were analyzed using either a paired or two sample student’s t-test as appropriate with equal variance not assumed (Welch Correction). The criterion for statistical significance was p < 0.05 and, unless otherwise noted in the text, p-values are as follows: *p < 0.05, **p < 0.01, and ***p < 0.001.

#### Cold plantar assay

To measure cold sensitivity of the hind paws, the cold plantar assay was performed as previously described^27,55^. Briefly, mice were allowed to acclimate in Plexiglas chambers for 2 hours prior to cold plantar testing performed at room temperature. A dry ice pellet was then applied to the hind paw of the mice through a glass at a thickness of 6 mm with a cutoff time of 20 sec to avoid tissue damage. Hind paw withdrawal latencies were recorded for a total of 3 trials per paw for each time point.

#### Hargreaves

To test heat sensitivity, the Hargreaves assay was performed^26,27^. Mice were allowed to acclimate in Plexiglas chamber on a glass plate heated to 32 °C for 30 min. Hind paw withdrawal latencies were measured. The basal paw withdrawal latency was adjusted to 9–12 s, with a cutoff time of 20 sec to avoid tissue damage. The paw withdrawal latency was averaged from 3 trials per paw.

#### Von frey

To determine mechanical sensitivity the up and down von Frey method was used^56^. Mice were placed on a mesh grid and a hindpaw was stimulated with graded von Frey filaments to determine the 50% withdrawal threshold.

### Isolation of mononuclear cells from the CNS

Isolation of mononuclear cells from spinal cord (SC) and brainstem (BS) and brain have been described previousl^57^. Briefly, two or three pooled BS or SC were minced and digested with collagenase and DNAse for 30 min after which the cell suspension was centrifuged through a two-step Percoll gradient at 800 g for 25 min at 4 °C. The resulting enriched population of viable mononuclear cells included CD45^high^ leukocytes, CD45^int^ microglia and CD45^neg^ BS resident glial cells (Supplementary Fig. 1). Cell viability was greater than 90% as revealed by trypan blue staining.

### Flow cytometery analysis

To determine cell surface expression, Ab-labeled cells were acquired on a BD Fortessa Analyzer (BD Biosciences, San Jose, CA) and flow cytometry analysis was performed using FlowJo software (Tree Star Inc.). Doublets were excluded from live cell populations. The gating strategy is shown in Supplementary Fig. 1. CD45 was used to distinguish BM-derived CD45^high^ leukocytes from CD45^int^ CD11b^+^ microglia and CD45^neg^ neural/glial cells. CD45^high^ monocytes/macrophages were determined by a SSC^low^ CD115^+^ CD11b^+^ Ly6G^−^ phenotype. The fold change in microglial populations within BS and SC of the different groups of mice treated with Paclitaxel compared to vehicle at day 5 after treatment is defined as the Microglial Index, shown in Supplementary Table 1.

### RT^2^ Prolfiler PCR Array Analysis of gene expression using Chemokine/Cytokine pathway and TLR pathway

Total RNA was isolated from homogenized and lysed SC samples of B6-B6mb and 129–129 mb mice at day 0 and day 5 post treatment (n = 3/group) using the RNeasy Mini Kit (Qiagen, Valencia, CA) and following genomic DNA elimination, cDNA synthesized from mRNA using the RT^2^ first strand kit (Qiagen), according to manufacturer’s instructions. Chemokine and Cytokine gene expression was analyzed using Syber Green based RT^2^ Profiler PCR Arrays (PAMM-011z for Inflammatory Cytokines & Receptors) in a 96 well plate format. RT-PCR was performed using manufacturers protocol and analysis performed using the manufacturer provided Analysis template. Briefly, data analysis was performed based on the ΔΔC_T_ method, with normalization of raw data (Gene of interest = GOI) to 3–5 housekeeping genes (HKG) that were not been altered following infection (<1.5 difference in C_T_ value between day 0 and day 6 pi). The ΔΔC_T_ value was calculated by subtracting the averaged ΔC_T_ value of the GOI at day 6 pi BS (test group) from the ΔC_T_ value of the day 0 BS (control group) and the fold change (up- or down- regulation) calculated as 2^(−ΔΔCT)^.

### Illumina 16S rRNA gene sequencing

Illumina bacterial 16S rRNA gene libraries were constructed as follows. PCRs were performed in an MJ Research PTC-200 thermal cycler (Bio-Rad Inc., Hercules, CA, USA) as 25 µl reactions containing: 50 mM Tris (pH 8.3), 500 µg/ml bovine serum albumin (BSA), 2.5 mM MgCl_2_, 250 µM of each deoxynucleotide triphosphate (dNTP), 400 nM of the forward PCR primer, 200 nM of each reverse PCR primer, 1 µl of DNA template, and 0.25 units JumpStart Taq DNA polymerase (Sigma-Aldrich, St. Louis, MO, USA). PCR primers 515 F (GTGCCAGCMGCCGCGGTAA) and 806 R (GGACTACHVGGGTWTCTAAT) were used to targeted the 16S rRNA gene containing portions of the hypervariable regions V4 and V5, with the reverse primers including a 12-bp barcode^58^. Thermal cycling parameters were 94 °C for 5 min; 35 cycles of 94 °C for 20 s, 50 °C for 20 s, and 72 °C for 30 s, and followed by 72 °C for 5 min. PCR products were purified using the MinElute 96 UF PCR Purification Kit (Qiagen, Valencia, CA, USA).

### Data processing

We used the UPARSE pipeline for de-multiplexing, length trimming, quality filtering and operational taxonomic units (OTU) picking using default parameters or recommended guidelines that were initially described in^59^ and which have been updated at https://www.drive5.com/usearch/manual/uparse_pipeline.html. Briefly, after demultiplexing, sequences were trimmed to a uniform length of 249 bp, then filtered at the recommended 1.0 expected error threshold, which kept approximately 90.3% of reads. Sequences were then dereplicated and clustered into zero-radius OTUs using the UNOISE3 algorithm^60^, which also detects and removes chimeric sequences; this method is based on making OTUs at 100% identity. An OTU table was then generated using the otutab command. OTUs having non-bacterial DNA were identified by performing a local BLAST search^61^ of their seed sequences against the nt database. OTUs were removed if any of their highest-scoring BLAST hits contained taxonomic IDs within Rodentia, Viridiplantae, Fungi, or PhiX. Taxonomic assignments to the OTUs were performed with SINTAX^62^ using RDP Classifier 16S training set number 16^63^ as the reference database.

### Microbiome data analyses

Beta diversity was measured using QIIME 1.9.1^64^ to calculate a Hellinger beta diversity distance matrix, which was depicted using principle coordinates analysis (PCoA), and statistically assessed by performing Adonis tests. Statistical differences among the OTUs were determined using edgeR^65,66^. Pearson correlation analyses were performed between bacteria relative abundances and pain values or microglia index values in R using the cor.test function and a proportion-normalized OTU table; the microglia index was calculated as decribed in supplementary Table 1. Heat maps were made using Heatmapper^67^ using the Expression function, average linkage clustering method, and Euclidean distance measurement method. OTU figures were made using Prism (GraphPad, La Jolla, CA). The bacterial sequences have been deposited in the National Center for Biotechnology Information (NCBI)’s Sequence Read Archive (SRA) under SRA Identifier Number SAMN11546114-SAMN11546431.

## Supplementary information

Supplementary Figures 1-6.
