# Supplementary material for: Dominant Role of the Gut Microbiota in Chemotherapy Induced Neuropathic Pain
Source: Sci Rep. 2019 Dec 30;9:20324. doi: 10.1038/s41598-019-56832-x (PMC6937259; doi:10.1038/s41598-019-56832-x)
Supplement: Supplementary file 1 — Supplementary Figures 1-6. [file 41598_2019_56832_MOESM1_ESM.pdf]

Supplementary Data.

Manuscript Title: Dominant Role of the Gut Microbiota in Chemotherapy Induced Neuropathic Pain.

Authors: Chandran Ramakrishna, Jose Corleto, Paul M. Ruegger, Geoffrey D. Logan, Beth B. Peacock, Stacee Mendonca, Shanni Yamaki, Trinka Adamson, Richard Ermel, David McKemy, James Borneman, and Edouard M. Cantin*.

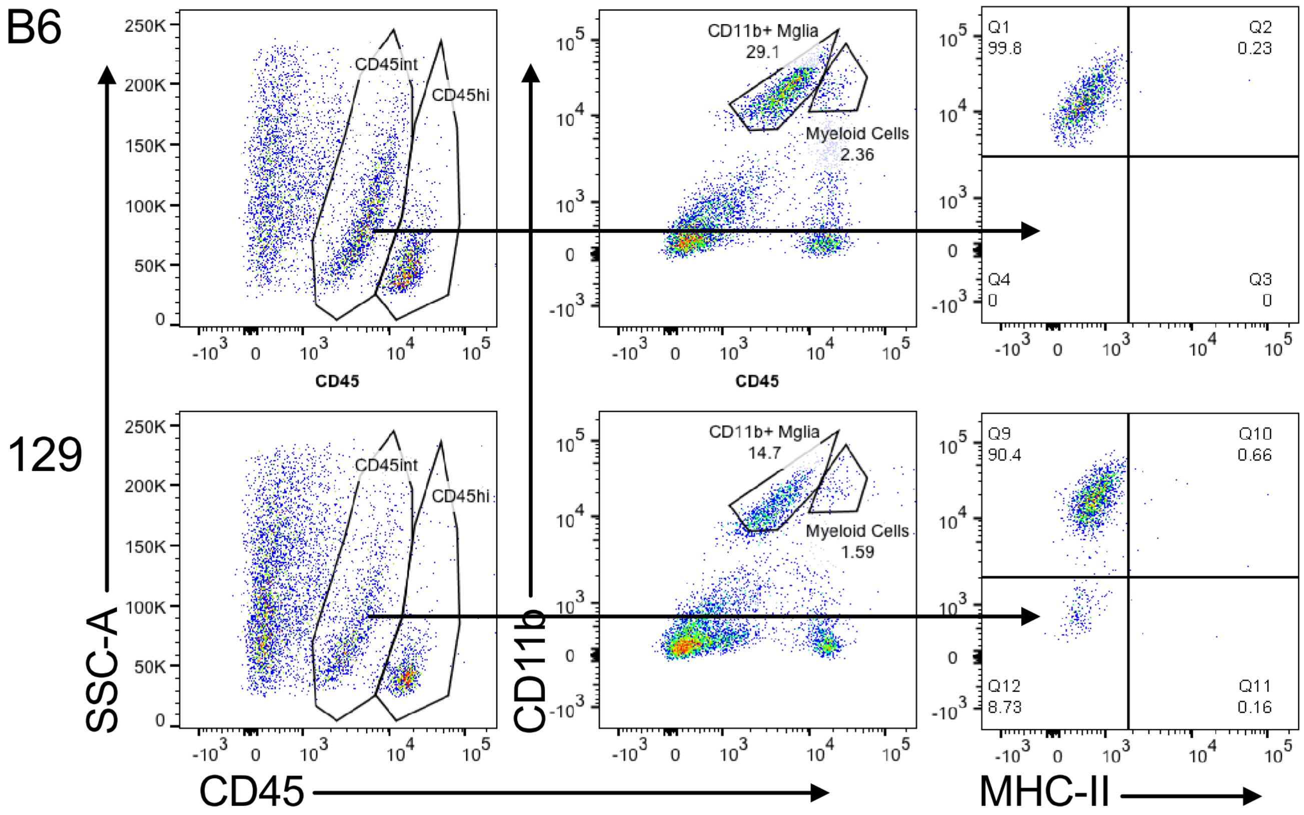
B6
129
SSC-A
CD45
CD11b
MHC-II
CD45int
CD45hi
CD11b+ Mglia
29.1
Myeloid Cells
2.36
Q1
99.8
Q2
0.23
Q4
0
Q3
0
CD11b+ Mglia
14.7
Myeloid Cells
1.59
Q9
90.4
Q10
0.66
Q12
8.73
Q11
0.16

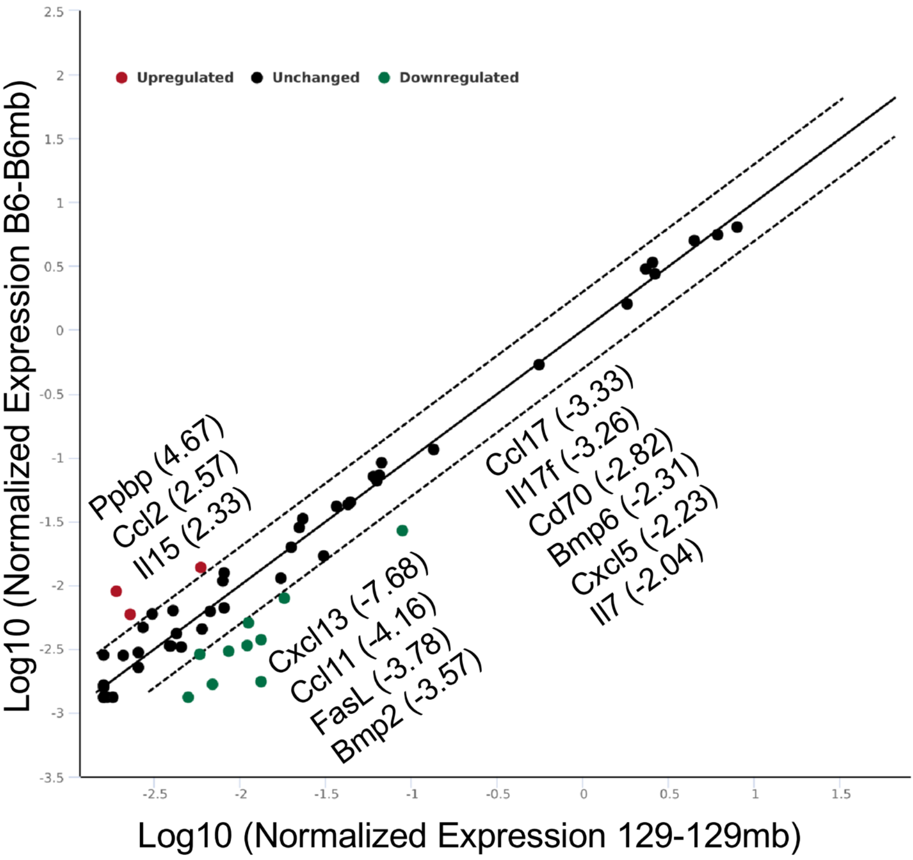

Upregulated
Unchanged
Downregulated
Ppbp (4.67)
Ccl2 (2.57)
Il15 (2.33)
Ccl17 (-3.33)
Il17f (-3.26)
Cd70 (-2.82)
Bmp6 (-2.31)
Cxcl5 (-2.23)
Il7 (-2.04)
Cxcl13 (-7.68)
Ccl11 (-4.16)
FasL (-3.78)
Bmp2 (-3.57)
Log10 (Normalized Expression B6-B6mb)
Log10 (Normalized Expression 129-129mb)
2.5
2
1.5
1
0.5
0
-0.5
-1
-1.5
-2
-2.5
-3
-3.5
-2.5
-2
-1.5
-1
-0.5
0
0.5
1
1.5

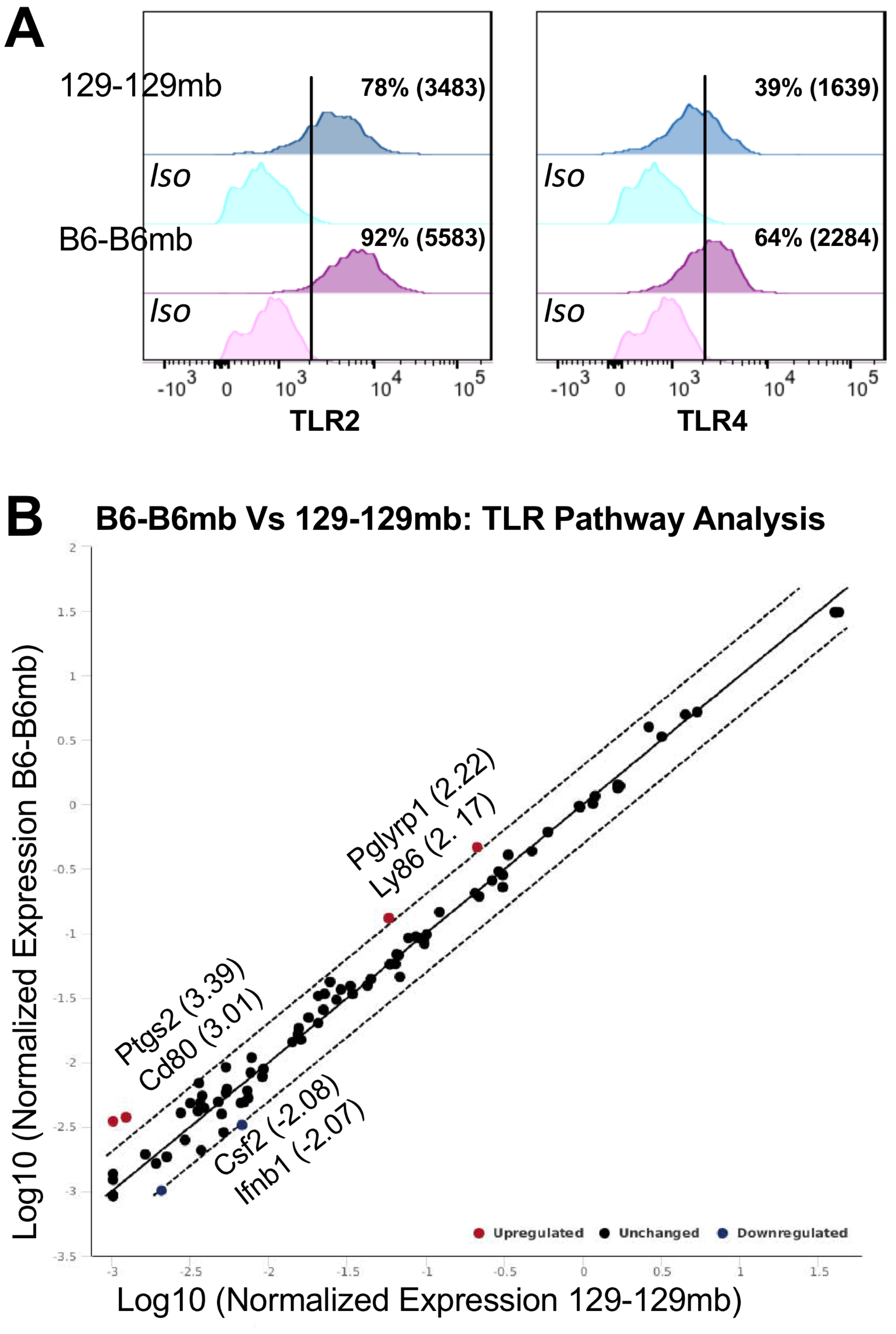
A
129-129mb
78% (3483)
Iso
B6-B6mb
92% (5583)
Iso
TLR2
39% (1639)
Iso
64% (2284)
Iso
TLR4
B
B6-B6mb Vs 129-129mb: TLR Pathway Analysis
Pglyrp1 (2.22)
Ly86 (2. 17)
Ptgs2 (3.39)
Cd80 (3.01)
Csf2 (-2.08)
Ifnb1 (-2.07)
Upregulated
Unchanged
Downregulated
Log10 (Normalized Expression B6-B6mb)
Log10 (Normalized Expression 129-129mb)

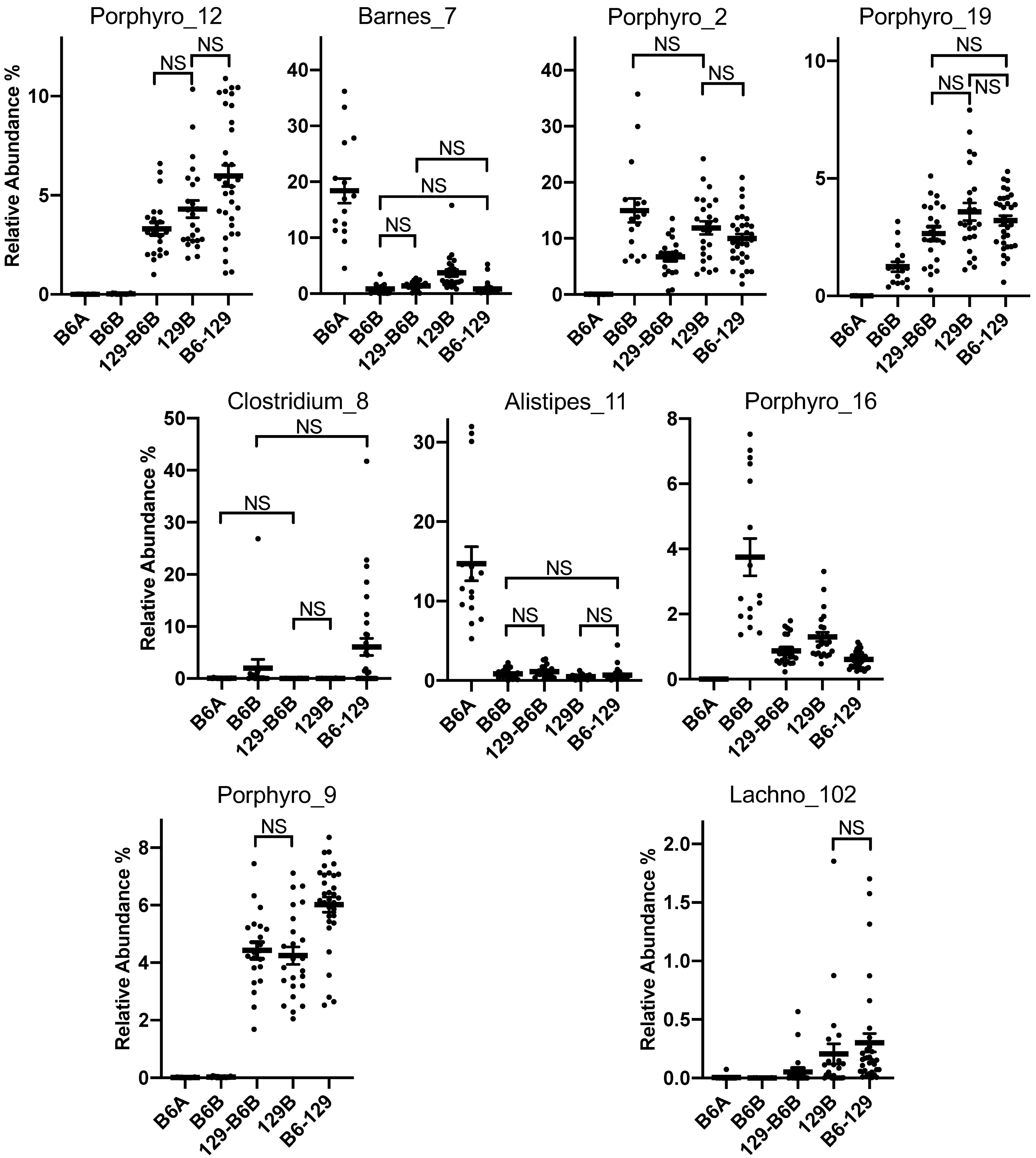

Porphyro_12
Barnes_7
Porphyro_2
Porphyro_19
Clostridium_8
Alistipes_11
Porphyro_16
Porphyro_9
Lachno_102
Relative Abundance %
NS
B6A
B6B
129-B6B
129B
B6-129

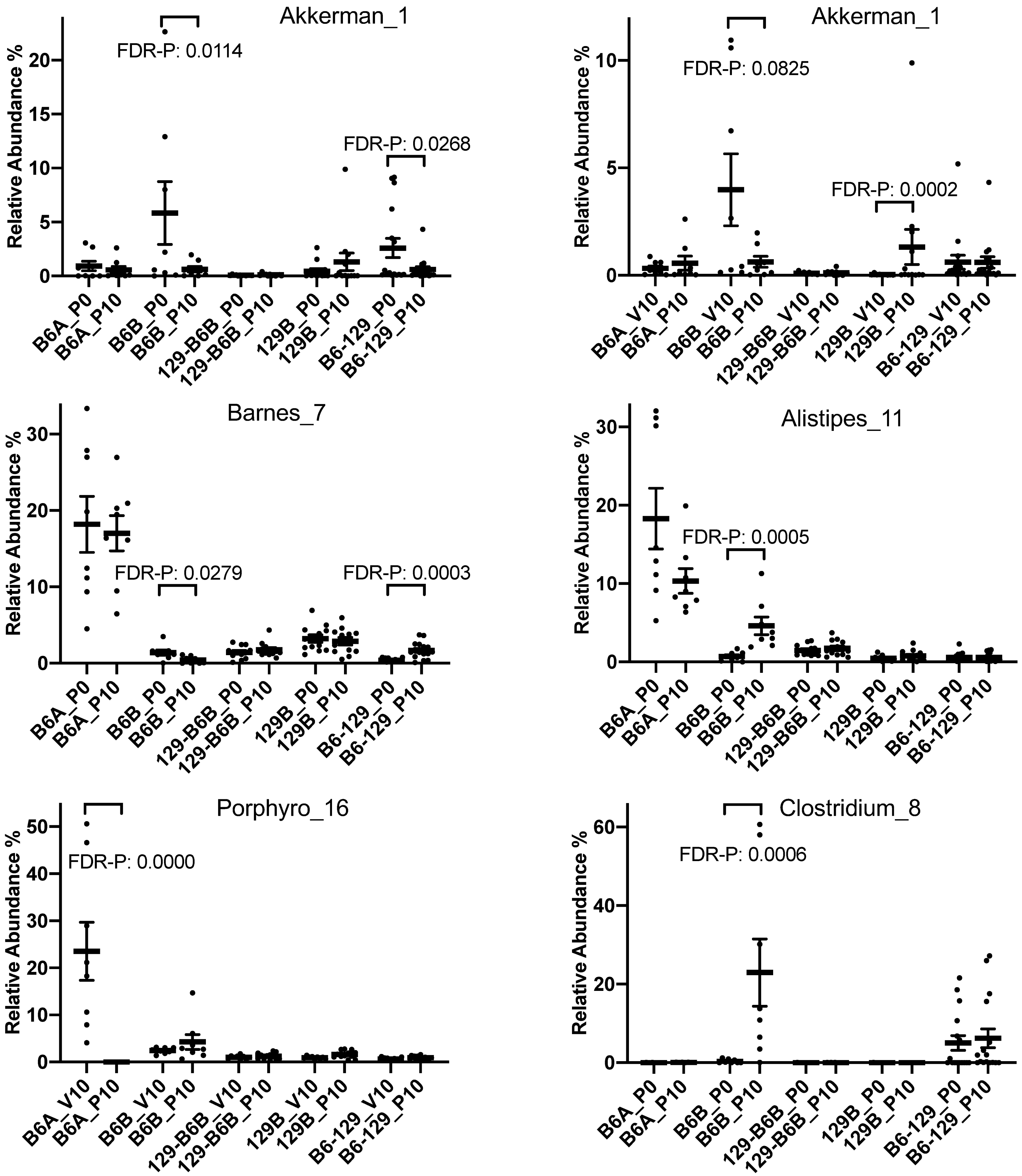
Akkerman_1
FDR-P: 0.0114
FDR-P: 0.0268
Relative Abundance %
0
5
10
15
20
B6A_P0
B6A_P10
B6B_P0
B6B_P10
129-B6B_P0
129-B6B_P10
129B_P0
129B_P10
B6-129_P0
B6-129_P10
Akkerman_1
FDR-P: 0.0825
FDR-P: 0.0002
Relative Abundance %
0
5
10
B6A_V10
B6A_P10
B6B_V10
B6B_P10
129-B6B_V10
129-B6B_P10
129B_V10
129B_P10
B6-129_V10
B6-129_P10
Barnes_7
FDR-P: 0.0279
FDR-P: 0.0003
Relative Abundance %
0
10
20
30
B6A_P0
B6A_P10
B6B_P0
B6B_P10
129-B6B_P0
129-B6B_P10
129B_P0
129B_P10
B6-129_P0
B6-129_P10
Alistipes_11
FDR-P: 0.0005
Relative Abundance %
0
10
20
30
B6A_P0
B6A_P10
B6B_P0
B6B_P10
129-B6B_P0
129-B6B_P10
129B_P0
129B_P10
B6-129_P0
B6-129_P10
Porphyro_16
FDR-P: 0.0000
Relative Abundance %
0
10
20
30
40
50
B6A_V10
B6A_P10
B6B_V10
B6B_P10
129-B6B_V10
129-B6B_P10
129B_V10
129B_P10
B6-129_V10
B6-129_P10
Clostridium_8
FDR-P: 0.0006
Relative Abundance %
0
20
40
60
B6A_P0
B6A_P10
B6B_P0
B6B_P10
129-B6B_P0
129-B6B_P10
129B_P0
129B_P10
B6-129_P0
B6-129_P10

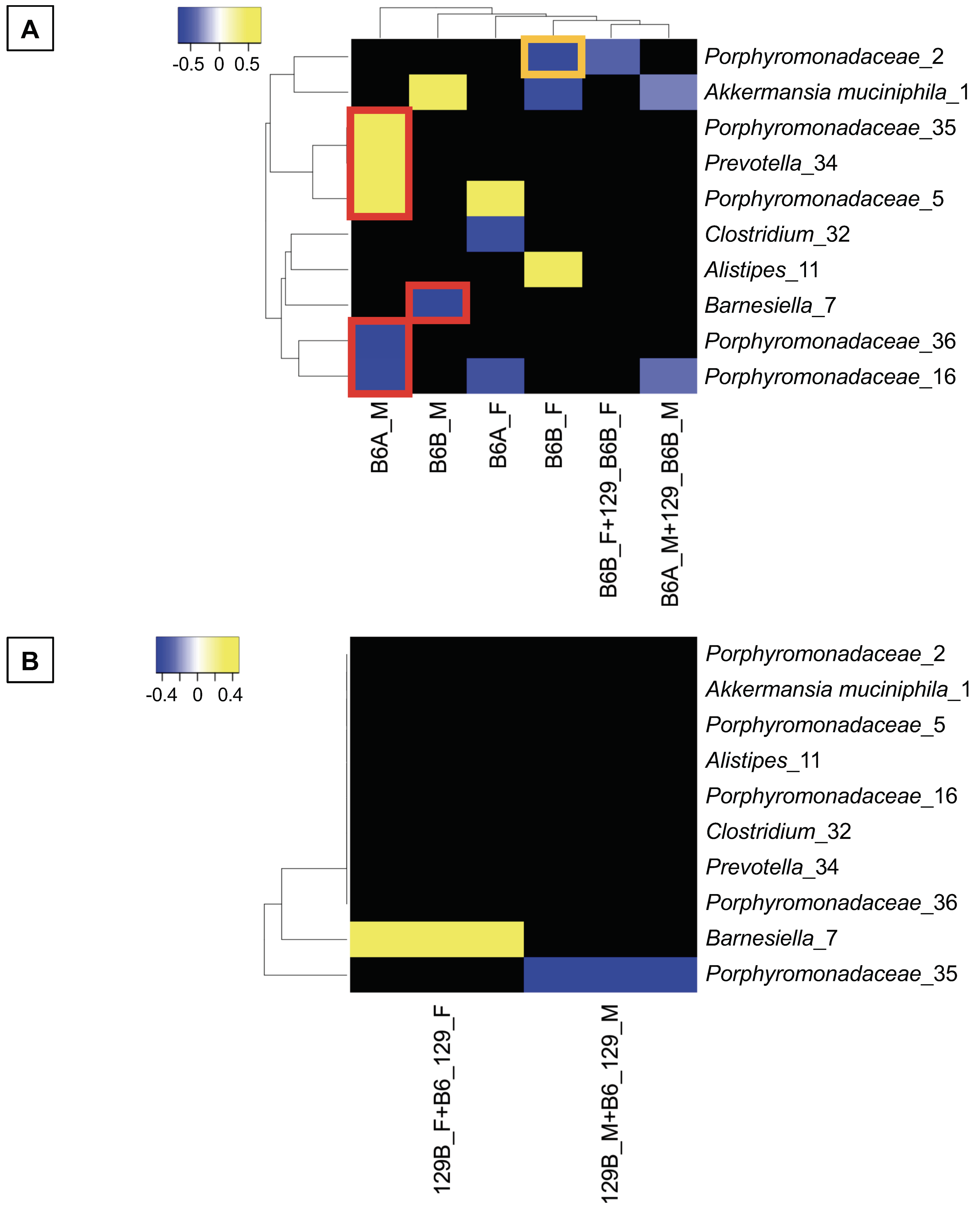

A
-0.5 0 0.5
Porphyromonadaceae_2
Akkermansia muciniphila_1
Porphyromonadaceae_35
Prevotella_34
Porphyromonadaceae_5
Clostridium_32
Alistipes_11
Barnesiella_7
Porphyromonadaceae_36
Porphyromonadaceae_16
B6A_M
B6B_M
B6A_F
B6B_F
B6B_F+129_B6B_F
B6A_M+129_B6B_M
B
-0.4 0 0.4
Porphyromonadaceae_2
Akkermansia muciniphila_1
Porphyromonadaceae_5
Alistipes_11
Porphyromonadaceae_16
Clostridium_32
Prevotella_34
Porphyromonadaceae_36
Barnesiella_7
Porphyromonadaceae_35
129B_F+B6_129_F
129B_M+B6_129_M

Supplementary Figure Legends:

Supplementary Figure 1: Gating Strategy for analysis of cells in the brainstem (BS) and spinal cords (SC). Mononuclear cells (singlets) isolated from the SC of WT B6 (top row) and 129 (bottom row) mice at day 5 post treatment were analyzed for CD45 expression and side scatter (left plot) or CD11b expression (middle plot); CD45^{low} gated microglial (MG) cells were analyzed for MHC II and CD11b (right plot).

Supplementary Figure 2: Minor differences in inflammatory cytokine and chemokine gene expression in SC of mice with B6 or 129 microbiome. Plot depicting inflammatory cytokine and chemokine genes upregulated in B6-B6mb mice (y-axis) or 129-129mb mice (x-axis); the cutoff value to determine upregulation was +2. Data taken from 3 mice / group.

Supplementary Figure 3: TLR expression in microglia in B6mb and 129mb mice. **(A)** Flow cytometry plots depicting expression of TLR2 (left plot) and TLR4 (right plot) on CD45^{low} MG of B6-B6mb and 129-129mb mice at day 5 post treatment (n=4 mice / group). Iso: Isotype. Numbers indicate % positive for TLR expression and numbers in parenthesis depict mean fluorescence intensity. **(B)** TLR pathway analysis was performed to determine genes that were different in the SC of B6-B6mb and 129-129mb at day 5 post treatment. Y-axis: B6-B6mb mice, X-axis: 129-129mb mice. A cutoff value of +2 was considered as significant for upregulation. Data compiled from n=3 mice / group.

Supplementary Figure 4. OTUs Whose Abundances Were Different Among the Genotype-MicrobiotaGroups at Day 0. In each of these figures, all possible pairwise comparisons of the genotype-microbiota groups are different (FDR-adjusted P value < 0.05) unless otherwise indicated by NS (not significant). These rules only apply to those pairs indicated by the vertical

lines of the brackets; for example, for Porphyro_9, the only pair that is not statistically significant is 129-B6B and 129B. Bacterial taxa abbreviations are: Alistipes, Alistipes onderdonkii; Barnes, Barnesiella; Clostridium, Clostridium aerotolerans; Lachno, Lachnospiraceae; Porphyro, Porphyromonadaceae; and the numbers following the underscores indicate the OTU numbers. Genotype-microbiota abbreviations are: B6-129, B6-129mb; 129-B6, 129-B6mbB. Means are the thick horizontal lines, error bars are standard error, and the replicate samples are the dots.

Supplementary Figure 5. Bacterial OTUs Whose Abundances Were Different Among the Genotype-Microbiota and Treatment Groups Due to Pac. The statistically significant comparisons are shown by brackets. Bacterial taxa abbreviations are: Akkerman, Akkermansia muciniphila; Porphyro, Porphyromonadaceae; Barnes, Barnesiella; Clostridium, Clostridium aerotolerans; Alistipes, Alistipes onderdonkii; and the numbers following the underscores indicate the OTU numbers. Genotype-microbiota and treatments abbreviations are: B6-129, B6-129mb; 129-B6, 129-B6mbB; P0, Pac at Day 0; P10, Pac at Day 10; V0, Veh at Day 0; V10, Veh at Day 10. Means are the thick horizontal lines, error bars are standard error, and the replicate samples are the dots.

Supplementary Figure 6. Correlations Between Bacteria and Microglia. Correlations between abundant fecal bacterial OTUs at Day 10 (Pac & Veh) in (A) B6mb groups or (B) 129mb groups and the microglia index. Values in cells are Pearson correlation coefficients; those shown have P-values < 0.05; those outlined in red or orange have FDR-adjusted Pvalues < 0.05 or equal to 0.127, respectively. Genotype-microbiota groups not shown had no correlations. Heat maps were clustered on both axes. Females: _F; Males _M. Numbers following the taxa indicate OTU numbers.
